# Analysis of tissue microstructure with Mueller microscopy: logarithmic decomposition and Monte Carlo modeling

**DOI:** 10.1117/1.JBO.25.1.015002

**Published:** 2020-01-13

**Authors:** Pengcheng Li, Hee Ryung Lee, Shubham Chandel, Christian Lotz, Florian Kai Groeber-Becker, Sofia Dembski, Razvigor Ossikovski, Hui Ma, Tatiana Novikova

**Affiliations:** a LPICM, CNRS, Ecole polytechnique, Institut Polytechnique de Paris, Palaiseau, France; b Tsinghua University, Department of Physics, Beijing, China; c Tsinghua–Berkeley Shenzhen Institute, Center for Precision Medicine and Healthcare, Shenzhen, China; d Graduate School at Shenzhen Tsinghua University, Institute of Optical Imaging and Sensing, Shenzhen Key Laboratory for Minimal Invasive Medical Technologies, Shenzhen, China; e Indian Institute of Science Education and Research, Department of Physical Sciences, Kolkata, India; f University Hospital Würzburg, Department of Tissue Engineering and Regenerative Medicine TERM, Würzburg, Germany; g Fraunhofer Institute for Silicate Research ISC, Translational Center Regenerative TherapiesLC-RT, Würzburg, Germany

**Keywords:** Mueller polarimetry, polarized Monte Carlo algorithm, scattering anisotropic media, skin tissue models, logarithmic decomposition, rotation invariants

## Abstract

**Significance**: Definitive diagnostics of many diseases is based on the histological analysis of thin tissue cuts with optical white light microscopy. Extra information on tissue structural properties obtained with polarized light would help the pathologist to improve the accuracy of his diagnosis.

**Aim:** We report on using Mueller matrix microscopy data, logarithmic decomposition, and polarized Monte Carlo (MC) modeling for qualitative and quantitative analysis of thin tissue cuts to extract the information on tissue microstructure that is not available with a conventional white light microscopy.

**Approach:** Unstained cuts of human skin equivalents were measured with a custom-built liquid-crystal-based Mueller microscope in transmission configuration. To interpret experimental data, we performed the simulations with a polarized MC algorithm for scattering anisotropic media. Several optical models of tissue (spherical scatterers within birefringent host medium, and combination of spherical and cylindrical scatterers within either isotropic or birefringent host medium) were tested.

**Results:** A set of rotation invariants for the logarithmic decomposition of a Mueller matrix was derived to rule out the impact of sample orientation. These invariants were calculated for both simulated and measured Mueller matrices of the dermal layer of skin equivalents. We demonstrated that only the simulations with a model combining both spherical and cylindrical scatterers within birefringent host medium reproduced the experimental trends in optical properties of the dermal layer (linear retardance, linear dichroism, and anisotropic linear depolarization) with layer thickness.

**Conclusions:** Our studies prove that Mueller polarimetry provides relevant information not only on a size of dominant scatterers (e.g., cell nuclei versus subwavelength organelles) but also on its shape (e.g., cells versus collagen fibers). The latter is directly related to the state of extracellular collagen matrix, which is often affected by early pathology. Hence, using polarimetric data can help to increase the accuracy of diagnosis.

## 1 Introduction

Probing the tissue with polarized light has been proven to be an efficient approach for fast noninvasive tissue diagnostics. Nevertheless, the interpretation of optical data and analytical prediction of the results of polarized light interaction with a biological tissue is not straightforward, as biological tissues are extremely complex objects containing a large number of randomly distributed multidisperse microscopic scatterers and anisotropic microstructures.^1^ It has been shown that Mueller matrix formalism is a powerful tool in the study of biological samples.^2^^–^^4^ This phenomenological approach describes the interaction of polarized light with a sample using a model of “equivalent optical circuit” built from the basic optical elements—diatenuators, retarders, and depolarizers. The extended toolkit of various Mueller matrix decompositions (data processing algorithms exploring nonlinear compression of a set of real values of 4×4 Mueller matrices) is available for data analysis and characterization of tissue optical properties.^5^^–^^10^ We have already proved the validity of the differential Mueller matrix formalism^9^^,^^10^ using Mueller transmission microscopy measurements of the dermal layer of histological cuts of skin equivalents,^11^^,^^12^ which were produced *in vitro* from human cells and accurately reflected the anatomy of human skin. Skin tissue models are widely employed as an alternative to animal models or human donor tissue. Since human skin equivalents can be produced with less variability compared with real human skin, these tissue models were chosen for our studies.

In this paper, we present the results of numerical modeling of optical properties of the dermal skin layer using various optical models and validate them with the experimental data of Lee et al.^12^ We use the Monte Carlo (MC) statistical algorithm for the solution of a vector radiative transfer equation and an appropriate optical model of tissue to simulate the propagation of polarized light through the biological sample. The polarized MC software for the modeling of scattering of polarized light on spherical and/or infinite-long cylindrical scatterers that are randomly distributed within either isotropic or uniaxial linear birefringent host medium was developed in prior studies.^13^^,^^14^ Each type of scatterer represents the different tissue components and microstructures (e.g., cell nuclei, cell organelles, extracellular collagen matrix, etc.). The optical model including both spheres and cylinders in birefringent medium [sphere-cylinder-birefringence (SCB) model] was previously tested on experimental data acquired from various biological samples.^15^^,^^16^ The SCB model predicted well the experimental trends in optical parameters extracted from the measured Mueller matrices.

It is known that values of some Mueller matrix elements and parameters extracted from Mueller matrix decomposition can be affected by the spatial orientation of a sample.^6^ Previously, a set of rotation invariants for Mueller matrix elements was proposed.^17^ In this study, we applied a logarithmic decomposition^9^ of both measured and simulated Mueller matrices and derived a set of invariant logarithmic Mueller matrix decomposition (LMMD) parameters to exclude the impact of sample orientation.

We have tested several optical models to reproduce the experimental dependence of the derived invariant parameters on tissue thickness. With our simulations, we ruled out the optical models of skin tissue that are based on (i) spheres and cylinders distributed in isotropic host medium [sphere-cylinder (SC) model] and (ii) spheres distributed in birefringent host medium model [sphere-birefringence (SB) model]. We found that only the optical model of the dermal layer, which includes both spheres and cylinders distributed in the birefringent host medium (SCB model), can reproduce the effects of anisotropic linear depolarization, linear dichroism, and linear retardance observed in our experiments. Moreover, we have confirmed that measured anisotropy of linear depolarization is a real effect that does not depend on sample orientation.

The trends in polarization and depolarization properties shown by the simulations qualitatively agree with the experiment results, thus, paving the way for use in optical biopsy, i.e., an understanding of the microstructure of biological samples from the polarimetric measurements.

## 2 Model Description

### 2.1 Monte Carlo Algorithm

The detailed description of the polarized MC algorithm and its implementation can be found in prior publications.^13^^,^^14^ Here, we recall the main steps of the statistical modeling approach while omitting some details for brevity. A point light source, placed at a fixed position in space, emits a given number ($10^{7}$ to $10^{8}$) of monoenergetic photons with the preset states of polarization. Those photons impinge a top surface of the sample at a given incident angle. Every photon travels a certain distance within the sample before being scattered on a sphere or cylinder. For each collision event, this distance is determined statistically using a mean free path parameter calculated from the scattering cross-sections of scatterers and their number density. The sizes, refractive indices, and number densities of sphere and cylinder scatterers as well as refractive index of isotropic host medium or ordinary and extraordinary refractive indices and spatial orientation of the optical axis of linear birefringent host medium are the input parameters of the optical model and can be adjusted to mimic the conditions of a real biological sample.^14^ The photon changes its polarization state and direction of propagation after each scattering event. The angles of deflection and rotation of polarization plane are calculated using the rejection method.^18^ A transfer matrix for scattering is determined by Mie theory for spherical scatterers or scattering matrix theory for infinite-long cylinder.^19^ The host medium may also be absorbing (not implemented in this study). The random walk of a photon continues within a scattering medium until it is either absorbed within the sample or moved outside the sample volume, where it can be lost or hit a detector. The first version of the software was developed on C language and run on a CPU platform.^13^ The latest version of the software is accelerated using a GPU platform.^20^

### 2.2 Optical Models of Skin Dermal Layer

To choose an appropriate optical model of a dermal layer of skin tissue cuts, one needs to account for both fibroblasts and well-aligned collagen fibers, which form the dermal equivalent of a skin model.^21^^,^^22^ While light scattering on cells and fibers produces depolarization, the optical anisotropy of the dermal layer results in retardance due to form birefringence.^1^ Thus, we used the monodisperse spherical scatterers in the optical model of the dermal layer to reproduce an isotropic scattering on cells. Infinitely long cylindrical scatterers were added to the optical model to simulate the effect of form birefringence due to the presence of aligned collagen fibers in dermis. The refractive indices of spherical and cylindrical scatterers ($n_{s}$, $n_{c}$) and isotropic medium ($n_{m}$) were set to 1.45 and 1.33, respectively.

We also explored the validity of replacing a form birefringence by an intrinsic birefringence of a uniaxial linear anisotropic host medium with in-plane optical axis, ordinary index $n_{m}^{o}=1.33$, and extraordinary index $n_{m}^{e}=1.33+\Delta n$, varying parameter Δn from $10^{-5}$ to $10^{-3}$. We took the values of refractive indices as for bulk fresh tissue,^1^ while noting that those values may be somewhat different for the studied fixed unstained tissue cuts. This is most probably not so important for our consideration because the refractive index of a scatterer and its size are highly correlated parameters in the Mie electromagnetic scattering problem. With our choice of refractive index values for both scatterers and host medium, the optical contrast $n_{s}/n_{m}$ (or $n_{c}/n_{m}$) is more than 1. Keeping constant the value of optical contrast, we varied the size of scatterers to reproduce the general trends in polarization and depolarization parameters in our simulations. We believe this is a reasonable assumption for performing the parametric numerical studies to reproduce the experimental trends.

In our experiments, a dermal layer of all skin model cuts demonstrated higher circular depolarization compared with the linear one ($\left|\alpha_{44}|>|\alpha_{22}\right|$, $\left|\alpha_{44}|>|\alpha_{33}\right|$). This suggests the dominance of the Rayleigh scattering regime over the Mie scattering regime^23^ and justifies the use of subwavelength spherical and cylindrical scatterers in an optical model of the dermal layer. The wavelength of probing light was fixed at 0.533  μm, so we tested spherical and cylindrical scatterers whose diameter ranged from 0.01 to 0.5  μm. Their concentrations $c_{s}$ and $c_{c}$ were described by the scattering coefficients $\mu_{s}$ and $\mu_{c}$ [$\mu_{s,c}=1/(c_{s,c}\sigma_{s,c})$, where $\sigma_{s,c}$ is the scattering cross-sections of sphere and cylinder, respectively), which were varied from 5 to $5000\;{\mathrm{cm}}^{-1}$. The parameter Δn for uniaxial birefringent host medium was adjusted to fit the experimental results^12^ for total linear retardance parameter $R_{T}$ (see Sec. 3.1). The optical axis of linear birefringent host medium was always oriented parallel to the sample surface, reflecting the arrangement of collagen fibers in a dermal layer of histological cuts. The GPU acceleration allowed us to carry out the simulations in a wide range of parameters to find the best-fit values.

Histological cuts of skin tissue models of varying thicknesses (nominal values 3 to 30  μm) were mounted on 1-mm-thick microscopy glass slides in our experiments.^12^ During the calibration of the Mueller polarimetric microscope with the eigenvalue calibration method,^24^ the measurements of air (one of the reference samples) were performed through a bare microscopy glass slide. Hence, the contribution of glass was excluded from Mueller matrices of all measured histological cuts. To model our experimental setup, we performed MC simulations in transmission configuration for the range of histological cut thicknesses defined by profilometer measurements^11^ without adding a 1-mm-thick glass layer to our optical model. A spatially uniform light beam was normally incident onto the flat front surface of a sample. No back surface roughness was taken into account in our optical model. The simulated images of forward scattering Mueller matrix elements were spatially averaged over a central circle of 600  μm in diameter to reproduce the experimental conditions, and the resulting Mueller matrices were decomposed using the LMMD method.^9^

## 3 Results and Discussion

### 3.1 Rotation Invariants of Logarithmic Decomposition

The set of polarization and depolarization parameters obtained from the LMMD includes the values of linear $\left(LB,LB^{\prime }\right)$ and circular (CB) retardance, linear $\left(LD,LD^{\prime }\right)$ and circular (CD) dichroism, and linear ($\alpha_{22}$, $\alpha_{33}$) and circular $\left(\alpha_{44}\right)$ depolarization coefficients.^9^ The parameters LB, CD, and $\alpha_{22}$ are defined with respect to the framework of 0 deg to 90 deg (linear polarization); the parameters $LB^{\prime }$, $LD^{\prime }$, and $\alpha_{33}$ are defined with respect to the framework of ±45  deg (linear polarization).

Neither optical activity nor circular dichroism was detected in the polarimetric measurement data for skin model histological cuts (CB=0, CD=0). The presence of well-aligned collagen fibers in a dermal layer of skin model cuts indicated the direction of the optical axis of uniaxial linear birefringent medium. In our experiments,^11^^,^^12^ the orientation of histological cuts in a sample holder plane was performed manually, thus, producing an uncertainty in azimuth of the optical axis from sample to sample. Therefore, the measured values of polarization parameters LB, $LB^{\prime }$ for skin model cuts of different thicknesses were affected not only by the different optical paths of the detected photons but also by the in-plane rotation of the samples. The experimental results for histological cuts of skin models have also demonstrated the existence of anisotropy of linear depolarization ($\alpha_{22}\neq \alpha_{33}$).^11^^,^^12^ Both parameters $\alpha_{22}$ and $\alpha_{33}$ are not invariant under the in-plane rotation of an anisotropic sample. Therefore, we also derived a rotation-invariant parameter for the linear retardance and linear depolarization anisotropy parameters to eliminate the impact of sample orientation with respect to the laboratory coordinate frame.

The logarithm of Mueller matrix M is calculated from the eigenvalue decomposition as

$$L=\ln\;M=\ln({U\Lambda U}^{-1})=U\;\ln(\Lambda )U^{-1},$$ (1)

where Λ is a diagonal matrix of eigenvalues of M and U is a matrix with the columns—eigenvectors of matrix M. The rotational transformation of a Mueller matrix in transmission configuration is described by $M^{\prime }=R(\alpha )\mathrm{MR}(-\alpha )$, where

$$R(\alpha )=[\begin{array}{cccc} 1 & 0 & 0 & 0\\ 0 & \cos(2\alpha ) & -\sin(2\alpha ) & 0\\ 0 & \sin(2\alpha ) & \cos(2\alpha ) & 0\\ 0 & 0 & 0 & 1 \end{array}].$$ (2)

Rotational transformation does not affect the eigenvalues of the matrix; therefore, we have

$$L^{\prime }=\ln\;M^{\prime }=\ln(R(\alpha ){U\Lambda U}^{-1}R(-\alpha ))=R(\alpha )U\;\ln(\Lambda )U^{-1}R(-\alpha )=R(\alpha )\mathrm{LR}(-\alpha ),$$ (3)

which means that the rotation transformation of matrix L is the same as for Mueller matrix M. As a result, the rotation invariants of matrix L should take the same form as the invariants for Mueller matrix M.^17^ If we denote $s_{n}=\sin(n\alpha )$ and $c_{n}=\cos(n\alpha )$ and decompose the matrix R(α)LR(−α) into the sum of polarization and depolarization matrices $L_{m}$ and $L_{u}$ (G-antisymmetric and G-symmetric components^9^), we get the following expressions:

$$L_{m}^{\prime }=\frac{1}{2}[\begin{array}{lllll} 0 & (L_{12}+L_{21})c_{2} & (L_{13}+L_{31})c_{2} & L_{14}+L_{41}\\ & \;-(L_{13}+L_{31})s_{2} & \;+(L_{12}+L_{21})s_{2} & \\ (L_{12}+L_{21})c_{2} & 0 & L_{23}-L_{32} & (L_{24}-L_{42})c_{2} & \\ \;-(L_{13}+L_{31})s_{2} & & & \;+(L_{43}-L_{34})s_{2}\\ (L_{13}+L_{31})c_{2} & L_{32}-L_{23} & 0 & (L_{34}-L_{43})c_{2}\\ \;+(L_{12}+L_{21})s_{2} & & & \;+(L_{24}-L_{42})s_{2}\\ L_{14}+L_{41} & (L_{42}-L_{24})c_{2} & (L_{43}-L_{34})c_{2} & 0\\ & \;+(L_{34}-L_{43})s_{2} & \;+(L_{42}-L_{24})s_{2} & \end{array}],$$ (4)

$$L_{u}^{\prime }=\frac{1}{2}[\begin{array}{llll} 2L_{11} & (L_{12}-L_{21})c_{2} & (L_{13}-L_{31})c_{2} & L_{14}-L_{41}\\ & \;+(L_{31}-L_{13})s_{2} & \;+(L_{12}-L_{21})s_{2} & \\ (L_{21}-L_{12})c_{2} & L_{22}+L_{33}+(L_{22}-L_{33})c_{4} & (L_{23}+L_{32})c_{4} & (L_{24}+L_{42})c_{2}\\ \;+(L_{13}-L_{31})s_{2} & \;-(L_{23}+L_{32})s_{4} & \;+(L_{22}-L_{33})s_{4} & \;-(L_{43}+L_{34})s_{2}\\ (L_{31}-L_{13})c_{2} & (L_{23}+L_{32})c_{4} & L_{22}+L_{33}+(L_{33}-L_{22})c_{4} & (L_{34}+L_{43})c_{2}\\ \;+(L_{21}-L_{12})s_{2} & \;+(L_{22}-L_{33})s_{4} & \;+(L_{23}+L_{32})s_{4} & \;+(L_{24}+L_{42})s_{2}\\ L_{41}-L_{14} & (L_{42}+L_{24})c_{2} & (L_{43}+L_{34})c_{2} & 2L_{44}\\ & \;-(L_{34}+L_{43})s_{2} & \;+(L_{42}+L_{24})s_{2} & \end{array}]$$ (5)

The rotation invariants of the matrix $L_{m}$ are total linear birefringence

$$R_{T}=\sqrt{L_{m42}^{2}+L_{m43}^{2}}=\sqrt{L_{m24}^{2}+L_{m34}^{2}}=\frac{1}{2}\sqrt{\left[{\left(L_{24}-L_{42}\right)}^{2}+{\left(L_{34}-L_{43}\right)}^{2}\right]},$$ (6)

total linear dichroism

$$D_{T}=\sqrt{L_{m12}^{2}+L_{m13}^{2}}=\sqrt{L_{m21}^{2}+L_{m31}^{2}}=\frac{1}{2}\sqrt{\left[{\left(L_{12}+L_{21}\right)}^{2}+{\left(L_{13}+L_{31}\right)}^{2}\right]},$$ (7)

circular birefringence

$$R_{C}=L_{m23}=-L_{m32}=(L_{23}-L_{32})/2,$$ (8)

and circular dichroism

$$D_{C}=L_{m14}=L_{m41}=(L_{14}+L_{41})/2.$$ (9)

Using the notation $\alpha_{ii}=L_{uii}$, (i=2,3,4) for the diagonal elements of matrix $L_{u}$, the rotation invariants of the matrix $L_{u}$ can be written in terms of linear (isotropic) depolarization

$$\alpha_{L}=(\alpha_{22}+\alpha_{33})/2=(L_{22}+L_{33})/2,$$ (10)

and circular depolarization

$$\alpha_{44}=L_{44}.$$ (11)

The four elements at the corners of the matrix L are also invariant under rotation, as well as the squared sum of the matrix elements from the first and last columns and the first and last rows. To find the rotation-invariant parameter for a linear anisotropic depolarization, we applied to matrix L the same procedure that was applied to matrix M in Mueller matrix transformation theory^17^ for defining a degree of anisotropy parameter $t_{1}=\frac{1}{2}\sqrt{{\left(m_{22}-m_{33}\right)}^{2}+{\left(m_{23}+m_{32}\right)}^{2}}$ and obtained

$$\alpha_{LA}=\frac{1}{2}\sqrt{{\left(\alpha_{22}-\alpha_{33}\right)}^{2}+{\left(L_{u23}+L_{u32}\right)}^{2}}.$$ (12)

In our optical model of tissue, the orientation of the optical axis of birefringence and the orientation of the axis of cylindrical scatterers were always along the X axis. In the experiments, the orientation of the aligned collagen fibers with respect to the edge of the microscope glass slide (i.e., laboratory X axis) depends on a sample preparation and may slightly vary from one histological cut to another. Therefore, we used the derived set of rotation invariants with nonzero values, namely, $R_{T}$, $D_{T}$ $\alpha_{L}$, $\alpha_{44}$, and $\alpha_{\mathrm{LA}}$ for the comparison of the results of measurements and simulations.

### 3.2 Choice of an Appropriate Optical Model of Dermis

Several optical models were tested to reproduce the optical effects observed in a dermal zone of samples. Some models (e.g., the SC model) were ruled out for the reasons discussed in Sec. 3.2.1. Finally, the SCB model was adopted in these studies, and the results are presented in Sec. 3.2.2.

#### 3.2.1 Ruled out optical models

Experimental studies of tissue phantoms^25^ and tissue models^12^ with transmission Mueller polarimetry had confirmed that retardance values calculated using LMMD depend linearly on thickness, while the depolarization parameters $\alpha_{22}$, $\alpha_{33}$, and $\alpha_{44}$, calculated using LMMD show quadratic dependence on layer thickness. To reproduce the experimental values of polarization and depolarization parameters of a dermal layer of histological cuts measured with the Mueller microscope in transmission configuration,^12^ we tested the SC, SB, and SCB optical models.

It was already demonstrated that at normal incidence an isotropic medium with spherical scatterers does not exhibit any retardance effect.^26^^,^^27^ A phase shift in the detected signal can be induced by scattering of polarized light by cylindrical scatterers as well as by light passing through a birefringent medium. Our modeling results demonstrated that the SC model with isotropic host medium fell short of reproducing the experimental values of retardance for low values of scattering coefficient $\mu_{c}$. The volume density (or concentration) of cylindrical scatterers had to increase significantly to fit the experimental trends in retardance, but with increase of parameter $\mu_{c}$ this model produced very high values of dichroism and depolarization which by far exceed the corresponding experimental values. Hence, we concluded that a uniaxial linear birefringent host medium is a necessary component of our optical model. It will increase the simulated retardance values without pushing up the dichroism and depolarization parameters of a simulated medium. Therefore, we ruled out the SC model for further consideration.

**Fig. 1:**
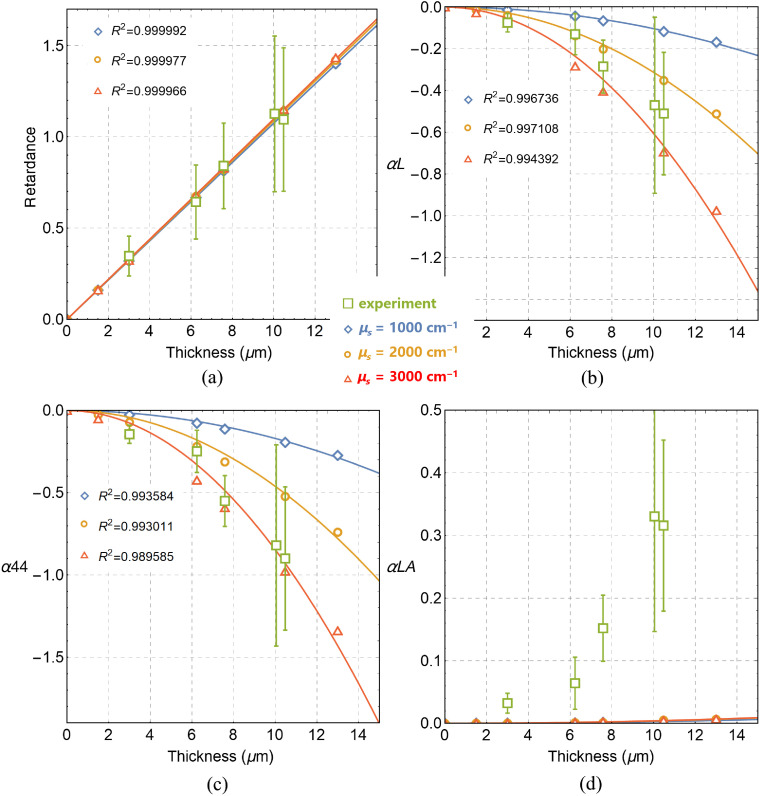
Results of MC simulations with the SB optical model for different layer thicknesses: (a) total linear retardance $R_{T}$ (radians) and dimensionless depolarization parameters (b) $\alpha_{L}$, (c) $\alpha_{44}$, and (d) $\alpha_{LA}$. Simulated data are shown by open symbols corresponding to different scattering coefficients (i.e., different concentrations of spherical scatterers). Open boxes with error bars represent the experimental data. Solid lines show the results of (a), (d) linear and (b), (c) quadratic fit of the simulated data.

We also examined the SB optical model of the skin dermal layer. The values of Δn parameter, radius of spherical scatterer $R_{s}$, and scattering coefficient $\mu_{s}$ were varied to find the best-fit to the experimental data. The SB optical model fits well the experimental values of retardance^11^ with optimal values of Δn=0.009 and $R_{s}=0.05\;\mu m$ [see Fig. 1(a)]. It is worth mentioning that the optimal value of Δn for the fixed tissue cuts was found to be about 2 orders of magnitude larger compared with the values reported for the fresh biological tissue.^1^ The simulation results with the SB optical model confirmed the linear dependence of total linear retardance $R_{T}$ (calculated from LMMD) on layer thickness. The simulated values of depolarization parameters $\alpha_{L}$, $\alpha_{44}$ have demonstrated a quadratic dependence on thickness within the experimental range of errors [see Figs. 1(b) and 1(c)]. No anisotropy of linear depolarization was observed with the SB model, as simulated values of $\alpha_{LA}=0$ for all layer thickness [Fig. 1(d)].

The experimental values of depolarization coefficients $\alpha_{22}$, $\alpha_{33}$, and $\alpha_{44}$ for a dermal layer of histological cuts obey the relation $\left|\alpha_{22}|<|\alpha_{33}|<|\alpha_{44}\right|$.^12^ However, the simulations with the SB model could not reproduce the effect of anisotropy of linear depolarization ($|\alpha_{LA}|\neq 0$) observed experimentally [see Fig. 1(d)]. Moreover, no linear dichroism can be simulated with the SB model, while the nonzero values of the linear dichroism were measured in our experiments.^12^ Therefore, we conclude that the optical SB model of the dermal layer of skin model histological cuts has to be modified to reproduce experimental trends.

#### 3.2.2 SCB model

We then added cylindrical scatterers to the optical model of dermis to simulate the effects of anisotropy of linear depolarization and linear dichroism. First, we used the same set of parameter values as for the SB optical model, but added a group of cylindrical scatterers of radius $R_{c}=0.05\;\mu m=R_{s}$, aligned along the X axis. The scattering coefficient for spherical scatterers $\mu_{s}$ was fixed at $1500\;{\mathrm{cm}}^{-1}$ and scattering coefficient for cylindrical scatterers $\mu_{c}$ was varied from 500 to $1500\;{\mathrm{cm}}^{-1}$.

**Fig. 2:**
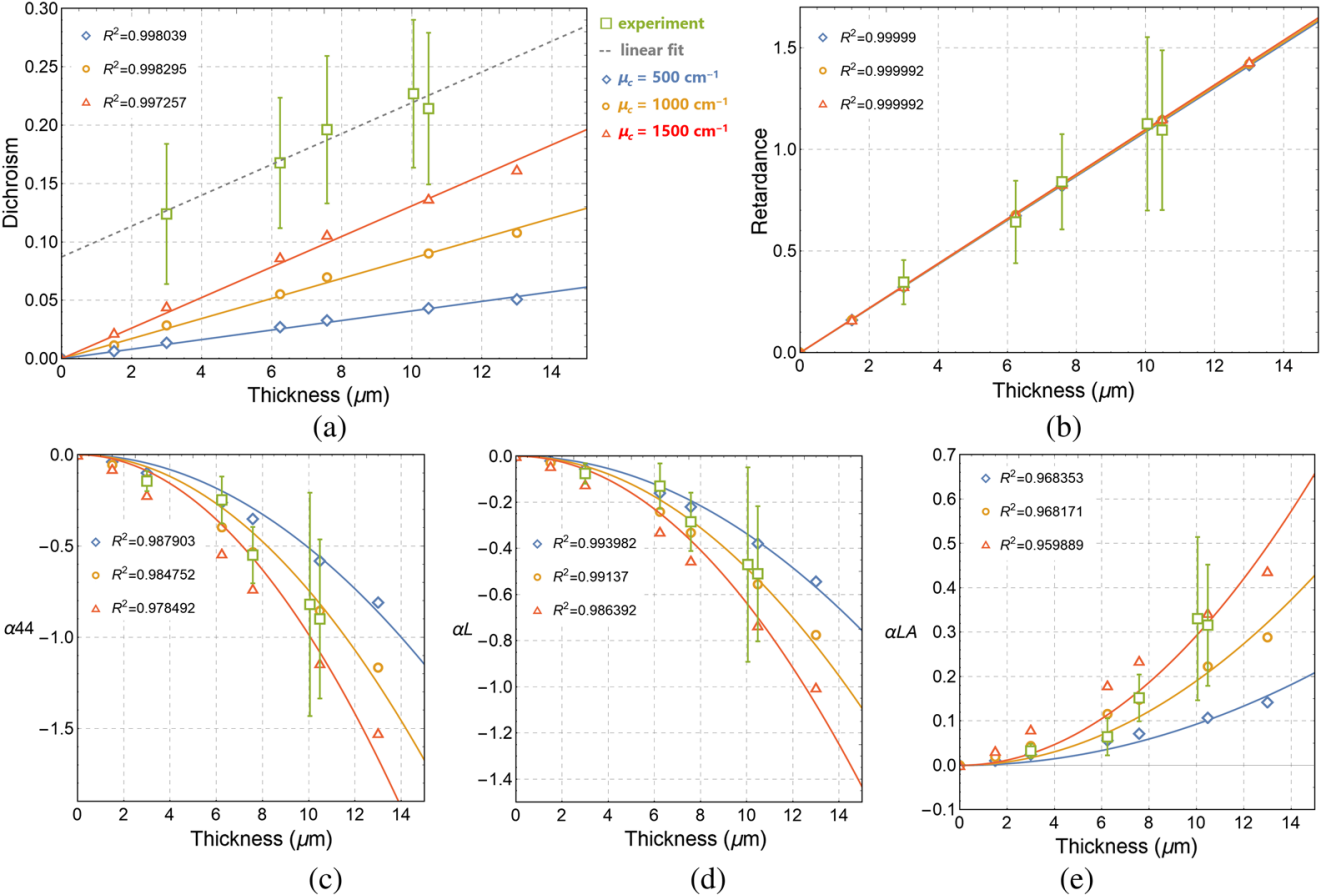
Results of MC simulations using the SCB optical model. Dependence on layer thickness of (a) total linear dichroism $D_{T}$ (dashed line is a linear regression curve for the experimental data), (b) total linear retardance $R_{T}$, (c)–(e) depolarization parameters $\alpha_{L}$, $\alpha_{44}$, and $\alpha_{LA}$, respectively. Simulated data are shown by open symbols corresponding to different scattering coefficients $\mu_{c}$ (i.e., different concentrations of cylindrical scatterers), $\mu_{s}=1500\;{\mathrm{cm}}^{-1}$, $R_{s}=R_{c}=0.05\;\mu m$. Open boxes with error bars represent the experimental data. Solid lines show the results of (a), (b) linear and (c)–(e) quadratic fit of the simulated data.

The results obtained with the SCB optical model after applying LMMD to the simulated Mueller matrices of layers of varying thicknesses are shown in Fig. 2. The presence of cylindrical scatterers had very limited influence on the values of linear retardance, but it had a significant impact on the values of linear dichroism and anisotropic depolarization effect. The SCB model yields the values of total linear retardance that also match well the experimental data [see Fig. 2(a)] The linear increase of total linear retardance $R_{T}$ and linear dichroism values on thickness are shown in Figs. 2(b) and 2(a), respectively. The nonzero intercept of linear regression curve for the experimental linear dichroism values with the Y axis [Fig. 2(b)] was explained by scattering of transmitted light on the rough surface of tissue.^12^ It was shown that for an anisotropic media a surface scattering does not affect the retardance values but has an impact on linear dichroism values.^28^ The quadratic dependence of $\alpha_{L}$, $\alpha_{44}$, and $\alpha_{LA}$ on thickness is also confirmed within the experimental range of errors. Moreover, an anisotropic depolarization effect ($|\alpha_{LA}|\neq 0$) is well reproduced with the SCB optical model [see Figs. 2(c)–2(e)].

**Fig. 3:**
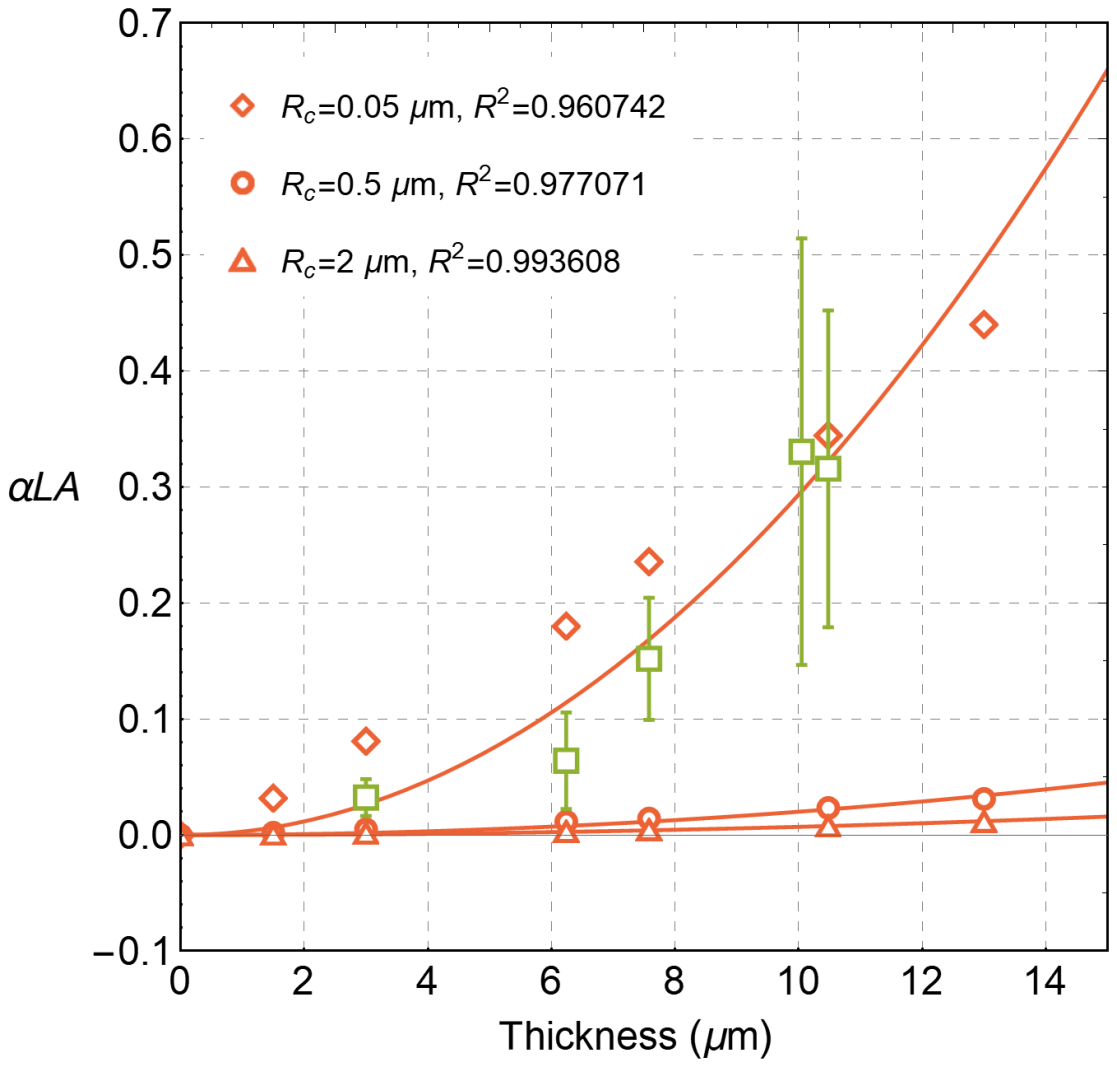
Dependence of parameter $\alpha_{LA}$ on layer thickness for different radii of cylindrical scatterers. The parameters of the SBC optical model are: $\mu_{s}=\mu_{c}=1500\;{\mathrm{cm}}^{-1}$, $R_{s}=0.05\;\mu m$. Open symbols correspond to the different radii of cylindrical scatterers: $R_{c}=0.05$, 0.5, and 2  μm, respectively. The concentration of cylindrical scatterers $c_{c}$ was adjusted to keep a constant value of the scattering coefficient $\mu_{c}$. Open boxes with error bars represent the experimental data. Solid lines show the results of a parabolic fit of the simulated data.

The impact of the radius of a cylindrical scatterer $R_{c}$ on anisotropic linear depolarization parameter $\alpha_{LA}$ was also studied (see Fig. 3). Our simulations show that cylinders with a smaller radius produce stronger anisotropy in linear depolarization. Hence, the presence of anisotropic linear depolarization is an indication of the scattering on small-size fibroid scatterers in the studied medium (so-called form birefringence). The values of parameter $\alpha_{LA}$ can be used for the estimation of the characteristic size of nonspherical scatterers.

To summarize, an appropriate optical model for a dermal layer of unstained fixed histological cuts of skin model tissue should include the subwavelength spherical scatterers and well-aligned cylindrical scatterers both distributed in a uniaxial linear birefringent medium. This model can qualitatively reproduce the thickness dependence of polarization and depolarization properties obtained from LMMD of the experimental Mueller matrices of a dermal layer of skin model histological cuts.^12^

## 4 Conclusions

We used MC software to model scattering of polarized light within the uniaxial birefringent scattering media and applied the logarithmic decomposition of simulated Mueller matrices to study a dependence of linear retardance, linear dichroism, and depolarization parameters on thickness in transmission configuration. Previously, it was confirmed that linear retardance and linear dichroism of the dermal layer of skin histological cuts depend linearly on thickness, while the depolarization varies quadratically with thickness. In these studies, we tested several different optical models to explain the results of transmission Mueller microscopy measurements of the histological cuts of full-thickness human skin equivalents. We found that the linear birefringence of the host medium is a necessary parameter of the optical model for reproducing the total linear retardance values, and anisotropic scatterers are the essential component of the optical model of dermis for reproducing both linear dichroism and anisotropic depolarization effects. We also derived the rotation-invariant parameters for LMMD and proposed using parameter $\alpha_{LA}$ as a marker for anisotropy of linear depolarization.

It was shown that measured values of linear retardance for a dermal layer of histological cuts can be simulated with either the SB or SCB model. Despite the fact that depolarization of transmitted light was reproduced with both optical models of dermis, the experimentally observed effect of anisotropy of linear depolarization ($\alpha_{LA}\neq 0$) required using the SCB optical model. Both skin tissue cuts measurements and simulations with the SCB optical model confirmed the presence of the nonzero linear dichroism calculated with LMMD for both measured and simulated Mueller matrices. The offset between experimental and simulated values of linear dichroism can be explained by the effect of light surface scattering, which was not included in our optical model. Extending the optical model of tissue by taking into account a rough interface between two media as well as testing different types of anisotropic scatterers (e.g., ellipsoids) will be the subject of future work.

With MC simulations, we have shown that applying the logarithmic decomposition of transmission Mueller matrix of tissue may provide the relevant information not only on average size of dominant scatterers but also on their shape. For example, the presence of the anisotropy of linear depolarization may point to the dominant scattering by nonspherical scatterers, thus, providing the information on tissue microstructure for optical diagnostics.
